# Association of hepatic/pancreatic iron overload evaluated by quantitative T2* MRI with bone mineral density and trabecular bone score

**DOI:** 10.1186/s12902-022-01262-6

**Published:** 2023-01-03

**Authors:** Zaizhu Zhang, Bo Hou, Guiying Du, Pengtao Sun, Wenmin Guan, Qiang Lin, Bing Han, Wei Yu

**Affiliations:** 1 Department of Radiology, Peking Union Medical College Hospital, Chinese Academy of Medical Science and Peking Union Medical College, No.1 Shuaifuyuan Wangfujing Dongcheng District, Beijing, 100730 China; 2 Department of Radiology, TEDA International Cardiovascular Hospital, Tianjin, China; 3 Department of Radiology, Beijing Shijitan Hospital, Capital Medical University, Beijing, China; 4 Department of Radiology, Beijing Friendship Hospital, Capital Medical University, Beijing, China; 5 Department of Radiology, Beijing Arion cancer center, Beijing, China; 6 Department of Hematology, Peking Union Medical College Hospital, Chinese Academy of Medical Sciences and Peking Union Medical College, Beijing, China

**Keywords:** Iron overload, T2*-value, Osteoporosis, Bone mineral density, Trabecular bone score

## Abstract

**Background:**

Iron-overloaded patients are recognized as presenting an increased risk of osteoporosis. However, studies on the correlation between osteoporosis and organ iron overload are controversial or scarce. The aim of this study is to assess bone mineral density (BMD) and trabecular bone score (TBS) in correlation with hepatic and pancreatic iron overload.

**Methods:**

Forty-one patients diagnosed with hemoglobinopathies, were studied. BMDs of the lumbar spine (LS), femoral neck (FN), and total hip (TH) were analyzed by Dual-energy X-ray absorptiometry (DXA) scan. LS bone quality was derived from each spine DXA examination using the TBS analysis. Hepatic and pancreatic iron overload were obtained with a multi-echo gradient echo T2* technique.

**Results:**

Abnormal microarchitecture and abnormal bone mass were observed in 19/41 (46.3%) and 9/41 (22.0%) patients, respectively. For 26 males, BMD, T-score and Z-score of LS were significantly lower among subjects with moderate-severe hepatic iron-overload than their counterparts, as it is between no- and pancreatic iron-overload groups. For 15 females, patients with moderate-severe hepatic iron-overload had significantly lower BMD and T-score of FN and TH, and patients with pancreatic iron-overload had significantly lower BMD, T-score of FN, and lower BMD, T-score and Z-score of TH than their counterparts. Moreover, pancreatic T2*-value was positively correlated with BMD and T-score at all analyzed sites and Z-score at TH.

**Conclusion:**

These data showed lower bone mass in patients with organ iron overload, particularly for LS in males, FN and TH in females. TBS may well represent a complementary tool for the evaluation of bone quality and the risk of fracture in iron-overloaded patients.

## Background

Iron overload is the result of many disorders that lead to excess iron deposition in the body, causing organ damage and increasing mortality [1]. Iron overload can be either primary, due to a deregulation of intestinal iron absorption as in hereditary hemochromatosis (HH), or secondary, resulting from ineffective erythropoiesis requiring long-term transfusions, including thalassemia, sickle cell disease and myelodysplastic syndrome (MDS), exogenous iron intake, or certain hematological diseases such as dyserythropoietic syndrome or chronic hemolytic anemia [1]. Iron overload may be asymptomatic, or may present with significant damage of the liver, heart, endocrine glands, joints, or other organs. Thus, assessment of organ iron concentration is vital and magnetic resonance imaging (MRI) has emerged as a non-invasive and popular alternative in view of the invasive nature of organ biopsy [2].

In addition to organ damage, iron overload could also lead to osteoporosis [3, 4], characterized by low bone mass and disturbed microarchitecture, resulting in increased bone fragility and a susceptibility to fractures [5]. Bone mineral density (BMD) determined by Dual-energy X-ray absorptiometry (DXA) remains the globally accepted “gold standard” method for noninvasive osteoporosis diagnosis [6]. However, the standard DXA test does not discriminate trabecular from cortical compartments and does not assess bone microarchitecture, hence decrease in BMD may only explain 60–70% of impairment in bone strength [7], and many fragility fractures occur in individuals with osteopenia or even normal BMD [8]. Trabecular bone score (TBS), as an emerging, noninvasive and complementary technology for evaluating bone quality, is a texture index that evaluates pixel gray-level variations in the lumbar spine DXA image [9]. TBS provides an indirect measure of bone microarchitecture and is highly correlated with direct assessments of the trabecular microarchitecture [10]. The role of TBS in predicting fracture risk, independently of BMD, has been demonstrated in postmenopausal and secondary osteoporosis [11]. However, studies that assess bone quality through TBS in patients with iron overload are scarce [12, 13]. Additionally, the results of the relationship between BMDs of different sites and organ iron overload are inconsistent and controversial [14–17].

Given this, we designed a cross-sectional study utilizing BMD and TBS to further investigate bone quantity and bone quality in patients with iron overload and their correlations with organ iron overload measured by MRI.

## Methods

### Study design and population

In this cross-sectional study, patients diagnosed with hemoglobinopathies, including HH, MDS, aplastic anemia, β-thalassemia, myelofibrosis, hereditary elliptocytosis and hereditary spherocytosis, were recruited, between March 2014 and May 2019. The key eligibility criteria were as follows: (1) age between 20 and 80 years, (2) body mass index (BMI) between 15 and 37 kg/m^2^, since the TBS analysis is not recommended in patients out of this BMI range [18]. Additionally, patients with any of the following conditions were excluded: (1) bone metabolic diseases such as definitive hypoparathyroidism and hyperparathyroidism; (2) rheumatic and neurological diseases; (3) pregnancy and lactation; (4) current or previous use of drugs that interfere with bone metabolism; (5) poor-quality DXA images. Then, the included patients underwent multi-echo gradient echo T2* MRI and DXA examinations on the same day for organ iron, BMD and TBS quantification.

### DXA measures

All DXA studies were performed on the same GE Lunar Prodigy Advance DXA scanner (GE Healthcare, Madison, WI), using the same software (enCORE version 10.50.086) for automatic calculation of BMD of the lumbar spine (LS), femoral neck (FN), and total hip (TH). According to WHO criteria, postmenopausal women and men aged 50 and older were classified as osteoporotic (T-score ≤ − 2.5), osteopenic (T-score between − 1.0 and − 2.5), and normal (T score ≥ − 1) [19]. According to the International Society for Clinical Densitometry (ISCD), premenopausal women and men younger than 50 years of age were defined as low bone mass (Z-score ≤ − 2.0) and normal (Z-score > − 2.0) [20].

TBS was assessed by TBS iNsight v2.1 software (Med-Imaps, Merignac, France) using the LS DXA images. Lumbar TBS was calculated as the mean value of individual measurements for vertebrae L1-L4. Accordingly, a TBS above 1.310 is considered normal (low risk fracture); a TBS between 1.230 and 1.310 is categorized as partially degraded microarchitecture (intermediate risk fracture); and a TBS below 1.230 is defined as degraded microarchitecture (high risk fracture) [21].

### MRI measures

All MRI examinations were performed on a 1.5 T MRI system (GE Excite HD), utilizing a single breath, multi-echo, fast gradient echo sequence. Relevant parameters were as follows: echo time = 2.0–11.8 ms; TE difference = 0.6 ms; repetition time = 200 ms; flip angle = 20°; field-of-view = 40 cm × 40 cm; matrix size = 128 × 96 pixels; slice thickness = 10 mm. The total acquisition time was about 17 s. The protocol used for T2* MRI measurements in all patients was based on the GE Advantage Workstation. The hepatic and pancreatic regions of interest (ROIs) were delineated by two experienced radiologists, and T2* was considered as the average of the several measurements. The hepatic and pancreatic iron contents were quantified in milliseconds (ms). Hepatic iron concentration values were set as follows: T2* > 11.4 ms indicated normal hepatic iron load; T2* of 3.8–11.4 ms indicated mild hepatic iron overload; and T2* of 1.8–3.8 ms indicated moderated iron overload, while severe hepatic iron overload was set at ≤ 2 ms. Taking the limited subjects in account, all patients were categorized into two groups based on hepatic cutoff points [22, 23]: no–mild iron-overload group (liver T2* ≥ 3.8 ms) and moderate–severe iron-overload group (liver T2* < 3.8 ms) and divided into two groups based on pancreatic cutoff points [22, 23]: no iron-overload group (pancreatic T2* ≥ 21 ms) and iron-overload group (pancreatic T2* < 21 ms), respectively.

### Statistical analysis

All statistical analyses were performed using SPSS 26.0 software (International Business Machines, Armonk, NY, USA). Categorical variables were reported as number (percentage) and assessed using Fisher’s exact test. Quantitative variables were tested for normality using the Shapiro–Wilk test. Normally and non-normally distributed continuous variables were expressed as the mean (standard deviation, SD) and median (interquartile range, IQR), and compared with Student’s t test and Mann–Whitney tests, respectively. Associations of iron-related parameters with TBS and BMD values were evaluated by Spearman’s correlation analysis. A *p* value < 0.05 was regarded as statistically significant.

## Results

### Subject characteristics

A total of 41 Chinese patients (26 men and 15 women; mean age ± SD, 47.54 years ±13.57) were recruited in this study, including 8 men aged 50 years or above and 8 postmenopausal women. Their clinical, laboratory and densitometric characteristics are shown in Table 1. The TBS of all subjects showed degraded microarchitecture in 6 (14.6%), partially degraded in another 13 (31.7%) and normal values in 22 (53.7%) patients. The patients with partially degraded and degraded microarchitecture were classified together as having abnormal microarchitecture (*n* = 19 (46.3%)). In contrast, LS osteoporosis (T-score ≤ − 2.5) and osteopenia (T-score ≤ − 1.0 or Z-score ≤ − 2.0) were observed in only one (2.4%) and 6 (14.6%) patients. A higher proportion of LS abnormality was found by TBS (19/41, 46.3%) than by BMD (7/41, 17.1%). Additionally, osteopenia was found at FN in 4 patients (9.8%) and at TH in 5 (12.2%). The patients with osteoporosis and osteopenia were classified together as having abnormal bone mass, which was found at the site with the lowest T-score/ Z-score in a total of 9 (22.0%) patients.

**Table 1:** Patient characteristics

Characteristics	Value
Age (years), mean (SD)	47.54 (13.57)
Sex, n (%)	
Male	26 (63.4)
≥ 50 years	8 (30.8)
Female	15 (36.6)
Postmenopause	8 (53.3)
BMI (kg/m^2^), mean (SD)	23.15 (3.04)
Serum ferritin (ng/mL), median (IQR)	1500.00 (1288.00)
Serum iron (μg/dL), mean (SD)	172.09 (81.41)
Liver T2*-value (ms), median (IQR)	4.80 (5.80)
No-mild iron overload (T2* ≥ 3.8 ms), n (%)	22 (53.7)
Moderate-severe iron overload (T2* < 3.8 ms), n (%)	19 (46.3)
Pancreatic T2*-value (ms), median (IQR)	35.20 (24.80)
No iron overload (T2* ≥ 21 ms), n (%)	31 (75.6)
Iron overload (T2* < 21 ms), n (%)	10 (24.4)
Primary disease, n. (%)	
Myelodysplastic syndrome	13 (31.7)
Aplastic anemia	14 (34.1)
Hereditary hemochromatosis	6 (14.6)
β-thalassemia	5 (12.2)
Myelofibrosis	1 (2.4)
Hereditary elliptocytosis	1 (2.4)
Hereditary spherocytosis	1 (2.4)
LS	
BMD (g/cm^2^), mean (SD)	1.08 (0.16)
T-score, mean (SD)	−0.20 (1.29)
Z-score, mean (SD)	0.38 (1.30)
Osteopenia, n (%)	6 (14.6)
Osteoporosis, n (%)	1 (2.4)
TBS, mean (SD)	1.33 (0.10)
Partially degraded microarchitecture, n (%)	13 (31.7)
Degraded microarchitecture, n (%)	6 (14.6)
FN	
BMD (g/cm^2^), mean (SD)	0.91 (0.12)
T-score, mean (SD)	− 0.39 (0.95)
Z-score, mean (SD)	0.13 (0.91)
Osteopenia, n (%)	4 (9.8)
Osteoporosis, n (%)	0 (0)
TH	
BMD (g/cm^2^), mean (SD)	0.95 (0.13)
T-score, mean (SD)	−0.29 (1.00)
Z-score, mean (SD)	0.03 (0.99)
Osteopenia, n (%)	5 (12.2)
Osteoporosis, n (%)	0 (0)
Abnormal bone mass, n (%)	9 (22.0)
Abnormal microarchitecture, n (%)	19 (46.3)

### Comparison of iron-related parameters, BMD and TBS between hepatic moderate-severe and no-mild iron overload groups

Among 26 male patients, BMD, T-score and Z-score of LS were significantly lower among subjects with moderate-severe hepatic iron overload compared to those with no-mild hepatic iron overload (1.00 ± 0.13 vs 1.14 ± 0.17, *p* = 0.027; − 0.81 ± 1.01 vs 0.37 ± 1.35, *p* = 0.022; − 0.49 ± 1.08 vs 0.66 ± 1.43, *p* = 0.035, respectively). TBS of LS and BMD, T-score and Z-score of FN and TH were all lower among subjects with moderate-severe hepatic iron overload compared to those with no-mild hepatic iron overload but not statistically significant. The moderate-severe hepatic iron overload group had significantly higher serum ferritin and iron levels than no-mild hepatic iron overload group (2183.67 ± 1475.39 vs 1018.14 ± 521.70, *p* = 0.048; 217.76 ± 95.88 vs 143.97 ± 56.44, *p* = 0.023, respectively). There was no significant difference in the mean age, BMI, pancreatic T2*-value and prevalence of abnormal bone mass and microarchitecture between two groups (Table 2).

**Table 2:** Comparison of iron-related parameters, BMD and TBS between hepatic moderate-severe and no-mild iron overload groups

	Male (*n* = 26)			Female (*n* = 15)		
	No-mild iron overload (*n* = 15)	Moderate-severe iron overload (*n* = 11)	*p*	No-mild iron overload (*n* = 7)	Moderate-severe iron overload (*n* = 8)	*p*
Age, mean (SD), years	43.20 (11.68)	45.18 (14.40)	0.702	48.14 (12.90)	58.38 (12.40)	0.144
Men aged 50 years or above/Women with postmenopause, n(%)	4 (26.7)	4 (36.4)	0.683	3 (42.9)	5 (62.5)	0.405
BMI, mean (SD), (kg/m^2^)	23.62 (2.69)	22.83 (2.47)	0.450	23.09 (1.98)	22.75 (5.06)	0.872
Serum ferritin (ng/mL), mean (SD)	1018.14 (521.70)	2183.67 (1475.39)	0.048	1070.57 (842.04)	3448.57 (2420.73)	0.042
Serum iron (μg/dL), mean (SD)	143.97 (56.44)	217.76 (95.88)	0.023	178.80 (101.80)	184.00 (31.55)	0.901
Liver T2*-value (ms), median (IQR)	9.71 (4.90)	1.96 (0.57)	0.000	6.22 (1.81)	1.94 (0.57)	0.000
Pancreatic T2*-value (ms), median (IQR)	42.30 (9.40)	20.00 (42.60)	0.160	38.27 (10.09)	23.26 (14.10)	0.041
BMD (g/cm^2^), mean (SD)						
Lumbar spine	1.14 (0.17)	1.00 (0.13)	0.027	1.09 (0.13)	1.07 (0.18)	0.872
Femoral neck	0.92 (0.13)	0.90 (0.07)	0.589	1.00 (0.13)	0.81 (0.08)	0.005
Total hip	0.96 (0.13)	0.91 (0.09)	0.318	1.09 (0.06)	0.91 (0.19)	0.043
T-score, mean (SD)						
Lumbar spine	0.37 (1.35)	−0.81 (1.01)	0.022	−0.24 (1.12)	−0.39 (1.43)	0.829
Femoral neck	−0.41 (1.02)	−0.57 (0.52)	0.608	0.20 (0.64)	−1.00 (0.63)	0.006
Total hip	−0.24 (0.98)	−0.59 (0.66)	0.342	0.88 (0.50)	−0.50 (1.44)	0.046
Z-score, mean (SD)						
Lumbar spine	0.66 (1.43)	−0.49 (1.08)	0.035	1.00 (0.98)	0.43 (1.15)	0.320
Femoral neck	0.01 (1.07)	−0.11 (0.65)	0.740	0.72 (0.67)	0.53 (0.54)	0.596
Total hip	−0.12 (0.97)	−0.43 (0.70)	0.398	0.98 (0.89)	0.40 (1.33)	0.350
Abnormal bone mass, n (%)						
Lumbar spine	0 (0.0)	2 (18.2)	0.169	2 (28.6)	3 (37.5)	1.000
Femoral neck	1 (6.7)	1 (9.1)	1.000	0 (0.0)	2 (25.0)	0.467
Total hip	1 (6.7)	1 (9.1)	1.000	1 (14.3)	2 (25.0)	1.000
TBS						
Lumbar spine, mean (SD)	1.36 (0.09)	1.33 (0.10)	0.540	1.33 (0.11)	1.28 (0.10)	0.415
Abnormal microarchitecture, n (%)	5 (33.3)	6 (54.5)	0.426	3 (42.9)	5 (62.5)	0.619

Among 15 female patients, patients with moderate-severe hepatic iron overload had significantly lower BMD of FN and TH (0.81 ± 0.08 vs 1.00 ± 0.13, *p* = 0.005 and 0.91 ± 0.19 vs 1.09 ± 0.06, *p* = 0.043, respectively) and lower T-score of FN and TH (− 1.00 ± 0.63 vs 0.20 ± 0.64, *p* = 0.006 and − 0.50 ± 1.44 vs 0.88 ± 0.50, *p* = 0.046, respectively). BMD, T-score, Z-score and TBS of LS, and Z-score of FN and TH were all lower among subjects with moderate-severe hepatic iron overload compared to those with no-mild hepatic iron overload but not statistically significant. The moderate-severe hepatic iron overload group had significantly higher serum ferritin and lower pancreatic T2*-value than no-mild hepatic iron overload group (3448.57 ± 2420.73 vs 1070.57 ± 842.04, *p* = 0.042; 23.26 ± 14.10 vs 38.27 ± 10.09, *p* = 0.041, respectively). Patients with moderate-severe hepatic iron overload did not differ significantly from those with no-mild hepatic iron overload in terms of age, BMI, serum iron levels and prevalence of abnormal bone mass and microarchitecture (Table 2).

### Comparison of iron-related parameters, BMD and TBS between pancreatic iron overload and no-iron overload groups

For 26 males, BMD, T-score and Z-score of LS were significantly lower among subjects with pancreatic iron overload compared to those without pancreatic iron overload (0.98 ± 0.12 vs 1.14 ± 0.16, *p* = 0.012; − 0.92 ± 0.95 vs 0.36 ± 1.32, *p* = 0.014; − 0.64 ± 0.89 vs 0.68 ± 1.44, *p* = 0.016, respectively). TBS of LS and BMD, T-score and Z-score of FN and TH were all lower among subjects with pancreatic iron overload compared to those without pancreatic iron overload but not statistically significant. The pancreatic iron overload group had significantly higher serum ferritin and iron levels and lower hepatic T2*-value than no iron-overload group (2875.63 ± 2082.68 vs 1080.63 ± 515.52, *p* = 0.045; 221.42 ± 101.30 vs 146.53 ± 55.14, *p* = 0.024; and 2.00 ± 1.30 vs 7.75 ± 8.10, *p* = 0.001, respectively). There were no significant differences in the mean age, BMI, and prevalence of abnormal bone mass and microarchitecture between two groups (Table 3).

**Table 3:** Comparison of iron-related parameters, BMD and TBS between pancreatic iron overload and no-iron overload groups

	Male (*n* = 26)			Female (*n* = 15)		
	No iron overload (*n* = 16)	iron overload (*n* = 10)	*p*	No iron overload (*n* = 10)	iron overload (*n* = 5)	*p*
Age, mean (SD), years	44.13 (11.70)	43.90 (14.75)	0.966	51.00 (11.99)	58.80 (15.74)	0.302
Men aged 50 years or above/Women with postmenopause, n(%)	5 (31.3)	3 (30.0)	1.000	5 (50.0)	3 (60.0)	0.573
BMI, mean (SD), (kg/m^2^)	23.34 (2.63)	23.20 (2.64)	0.898	23.69 (3.79)	21.34 (3.72)	0.276
Serum ferritin (ng/mL), median (IQR)	1080.63 (515.52)	2875.63 (2082.68)	0.045	1619.67 (1103.58)	2315.75 (2246.10)	0.457
Serum iron (μg /dL), mean (SD)	146.53 (55.14)	221.42 (101.30)	0.024	178.34 (86.06)	147.75 (86.43)	0.560
Liver T2*-value (ms), median (IQR)	7.75 (8.10)	2.00 (1.30)	0.001	4.85 (4.20)	2.00 (4.50)	0.440
Pancreatic T2*-value (ms), median (IQR)	43.00 (12.10)	7.65 (5.20)	0.000	37.60 (15.3)	7.60 (12.40)	0.001
BMD (g/cm^2^), mean (SD)						
Lumbar spine	1.14 (0.16)	0.98 (0.12)	0.012	1.12 (0.14)	1.01 (0.16)	0.179
Femoral neck	0.94 (0.15)	0.91 (0.08)	0.646	0.99 (0.13)	0.79 (0.08)	0.011
Total hip	0.97 (0.16)	0.93 (0.11)	0.503	1.07 (0.11)	0.79 (0.10)	0.000
T-score, mean (SD)						
Lumbar spine	0.36 (1.32)	−0.92 (0.95)	0.014	0.00 (1.18)	−0.95 (1.26)	0.172
Femoral neck	−0.29 (1.22)	−0.49 (0.63)	0.647	0.43 (1.10)	−1.15 (0.69)	0.012
Total hip	−0.11 (1.26)	− 0.44 (0.85)	0.477	0.72 (0.82)	−1.42 (0.77)	0.000
Z-score, mean (SD)						
Lumbar spine	0.68 (1.44)	−0.64 (0.89)	0.016	0.86 (1.14)	0.49 (0.95)	0.550
Femoral neck	0.18 (1.35)	−0.05 (0.70)	0.623	1.18 (1.19)	0.09 (1.14)	0.113
Total hip	0.03 (1.29)	−0.31 (0.84)	0.480	1.22 (0.81)	−0.44 (0.91)	0.003
Abnormal bone mass, n (%)						
Lumbar spine	0 (0.0)	2 (20.0)	0.138	2 (20.0)	3 (60.0)	0.251
Femoral neck	1 (6.3)	1 (10.0)	1.000	0 (0.0)	2 (40.0)	0.095
Total hip	1 (6.3)	1 (10.0)	1.000	0 (0.0)	3 (60.0)	0.022
TBS						
Lumbar spine, mean (SD)	1.35 (0.10)	1.34 (0.08)	0.614	1.32 (0.11)	1.29 (0.11)	0.601
Abnormal microarchitecture, n (%)	5 (31.3)	6 (60.0)	0.228	5 (50.0)	3 (60.0)	1.000

For 15 females, patients with pancreatic iron overload had significantly lower BMD of FN and TH (0.79 ± 0.08 vs 0.99 ± 0.13, *p* = 0.011 and 0.79 ± 0.10 vs 1.07 ± 0.11, *p* = 0.000, respectively), lower T-score of FN and TH (− 1.15 ± 0.69 vs 0.43 ± 1.10, *p* = 0.012 and − 1.42 ± 0.77 vs 0.72 ± 0.82, p = 0.000, respectively), and lower Z-score of TH (− 0.44 ± 0.91 vs 1.22 ± 0.81, *p* = 0.003). The prevalence of abnormal bone mass at TH was significantly higher in the pancreatic iron overload group than in the no iron-overload group (60.0% vs 0%, *p* = 0.022). BMD, T-score, Z-score and TBS of LS, and Z-score of FN were all lower among subjects with pancreatic iron overload compared to those without pancreatic iron overload but not statistically significant. Patients with pancreatic iron overload did not differ significantly from those without pancreatic iron overload in terms of age, BMI, serum ferritin and iron levels, hepatic T2*-value and prevalence of abnormal bone mass at LS and FN and abnormal microarchitecture at LS (Table 3).

### Correlations among iron-related parameters, BMD and TBS

For the correlations among iron-related parameters in both male and female individuals, liver T2*-value was positively correlated with pancreatic T2*-value (*r* = 0.471; *P* = 0.002) and negatively correlated with serum ferritin (*r* = − 0.592; *P* = 0.000) and serum iron (*r* = − 0.413; *P* = 0.009), respectively. Pancreatic T2*-value showed significant negative correlation with serum ferritin (*r* = − 0.374; *P* = 0.021). For the correlations among liver T2*-value, BMD and TBS values, no significant correlation was found. However, spearman’s correlation analysis demonstrated a significant positive correlation between pancreatic T2*-value with BMD and T-score at all analyzed sites and Z-score at TH: LS BMD (*r* = 0.357, *P* = 0.022); FN BMD (*r* = 0.399, *P* = 0.010); TH BMD (*r* = 0.432, *P* = 0.005); LS T-score (*r* = 0.347, *P* = 0.026); FN T-score (*r* = 0.402, *P* = 0.010); TH T-score (*r* = 0.464, *P* = 0.003); TH Z-score (*r* = 0.381, *P* = 0.015), but neither LS Z-score, FN Z-score nor TBS were correlated with pancreatic T2*-value (Table 4).

**Table 4:** Correlations among iron-related parameters, BMD and TBS

	Liver T2*-value		Pancreatic T2*-value	
	*r* Value	*P* Value	*r* Value	*P* Value
Liver T2*-value	/	/	0.471	0.002
Pancreatic T2*-value	0.471	0.002	/	/
Serum ferritin (ng/mL)	−0.592	0.000	−0.374	0.021
Serum iron (μg /dL)	−0.413	0.009	−0.201	0.221
BMD (L1–4)	0.208	0.191	0.357	0.022
BMD (Femoral neck)	0.139	0.385	0.399	0.010
BMD (Total hip)	0.165	0.303	0.432	0.005
T-score (L1–4)	0.205	0.198	0.347	0.026
T-score (Femoral neck)	0.126	0.439	0.402	0.010
T-score (Total hip)	0.152	0.350	0.464	0.003
Z-score (L1–4)	0.108	0.501	0.273	0.084
Z-score (Femoral neck)	0.020	0.905	0.295	0.065
Z-score (Total hip)	−0.001	0.994	0.381	0.015
TBS (L1–4)	0.024	0.883	0.021	0.898

## Discussion

To our knowledge, this is the first cross-sectional study to evaluate the correlations of BMD, TBS and organ iron overload measured by MRI in iron-overloaded patients. In the study, abnormal bone mass was found at any of the three sites in a total of 9 (22.0%) patients, of which 7 patients had LS abnormality. Notably, 19 (46.3%) patients had abnormal microarchitecture by TBS, which implied that TBS may be a more sensitive parameter than BMD to assess vertebral bone strength in iron-overloaded patients. Moreover, because TBS uses previously obtained DXA images, it is simple to use in routine practice, enables the generation of longitudinal data concerning cross-sectional and clinical studies and allows direct comparison with areal BMD and application to existing datasets [24]. Hence, the authors propose TBS be used as a valuable and complementary clinical tool in the general diagnosis of osteoporosis and in fracture risk assessment in iron-overloaded patients. From a clinical perspective, the additional information about bone strength provided by TBS could aid the management of patients with osteopenia, as well as those with normal BMD who have risk factors for osteoporosis.

In the study, TBS of LS had no significant differences between moderate-severe and no-mild hepatic iron overload groups, as it is between no- and pancreatic iron overload groups. Neither hepatic nor pancreatic T2*-value was correlated with LS TBS, in line with the result by Baldini et al. [12] that TBS did not correlate with liver iron concentration values. However, the studies by Baldini et al. [12] and Banaszkiewicz et al. [13] revealed that TBS was significantly lower in patients with iron overload than controls. It should be emphasized that although not statistically significant in the study, TBS values in the moderate-severe hepatic and pancreatic iron overload groups were lower than in their counterparts, which demonstrated that organ iron overload may have a correlation with disturbed bone microarchitecture. The absence of statistical significance in our groups may be due to the limited number of events, a hypothesis to be verified by enlarging the population studied.

As is well known, hepatic iron concentration (HIC) is considered the surrogate marker of total body iron stores [2]. In the study, we observed that BMD, T-score and Z-score of the three sites were lower among subjects with moderate-severe hepatic iron overload compared to those with no-mild hepatic iron overload, of which BMD, T-score and Z-score of LS in males and BMD, T-score of FN and TH in females had statistical significance. This result suggests that hepatic iron overload may lead to decreased BMD and increased risk of osteoporosis. In accordance with this result, HIC has been reported to be significantly higher in patients with osteoporosis than in patients without osteoporosis [25]. Moreover, in a study of 38 male HH patients, a negative correlation was found between HIC and BMD at the FN [16].

Many studies have reported that iron overload could cause endocrine dysfunction, which in turn lead to osteoporosis [26, 27]. Pancreas is one of the most important endocrine organs, hence, it is highly probable that pancreatic iron overload is closely related to osteoporosis. The study showed that BMD, T-score and Z-score of the three sites were lower among subjects with pancreatic iron overload compared to those without pancreatic iron overload, of which BMD, T-score and Z-score of LS in males and BMD, T-score of FN and BMD, T-score and Z-score of TH in females had statistical significance. Moreover, the prevalence of abnormal bone mass at TH in females was significantly higher in the pancreatic iron overload group than in the no iron-overload group. There were significant positive correlations between pancreatic T2*-value with BMD and T-scores at all analyzed sites and Z-scores at TH. Our findings demonstrated that compared with liver, pancreatic iron overload may be more closely related to bone loss and the occurrence of osteoporosis.

Interestingly, LS in males, FN and TH in females are more prone to the decrease in bone mass in both subgroups. Similarly, Valenti et al. [14], in a study of 17 female and 70 male patients with HH, found that BMD was lower in the LS compared to the FN, either evaluated by the T-score or the Z-score. However, another study by Guggenbuhl et al. [16] found that in 38 male HH patients, the decrease in BMD was more pronounced at the FN than at the LS. Therefore, the effect of gender on the osteoporosis of different sites needs more attention and further research.

Additionally, our study, in parallel with previous reports [28–31], indicated that liver T2*-value was significantly correlated with pancreatic T2*-value, serum ferritin and serum iron and pancreatic T2*-value showed significant negative correlation with serum ferritin, whereas others have shown no correlation between hepatic and pancreatic iron [32, 33]. These controversial results may be attributed to differences in the type of disease, number of subjects, and management.

A limitation of this study is that the relatively small sample size in our study may result in biased results, particularly for females. Age and menopausal status may make a difference in bone health, so further research is needed to confirm the conclusions of this study. A further limitation is that we could not exclude the possibility that iron would spill over into the bone marrow to increase LS BMD measured by DXA in iron-overloaded patients, particularly in cases of moderate–severe iron overload.

## Conclusions

This study confirmed that bone mass of all three sites seems lower in patients with hepatic moderate-severe and/or pancreatic iron overload, particularly for LS in males, FN and TH in females. Moreover, TBS may well represent a complementary tool for the evaluation of bone quality and the risk of fracture in iron-overloaded patients.

## Data Availability

The datasets generated and/or analysed during the current study are not publicly available due [REASON WHY DATA ARE NOT PUBLIC] but are available from the corresponding author on reasonable request.

## Abbreviations

BMD: Bone mineral density
TBS: Trabecular bone score
LS: Lumbar spine
FN: Femoral neck
TH: Total hip
DXA: Dual-energy X-ray absorptiometry
HH: Hereditary hemochromatosis
MDS: Myelodysplastic syndrome
MRI: Magnetic resonance imaging
BMI: Body mass index
ISCD: The International Society for Clinical Densitometry
ROIs: Regions of interest
ms: Milliseconds
SD: Standard deviation
IQR: Interquartile range
HIC: Hepatic iron concentration
